# Address

**Published:** 1878-05

**Authors:** James Taylor

**Affiliations:** President of the Board of Trustees


### ADDRESS.

*By Dr. James Taylor, President of the Board of Trustees, upon conferring the degree of D. D. S. upon the members of the Graduating Class in the Ohio College of Dental Surgery, March 6th, 1878.*

Gentlemen Graduates:—Allow me to congratulate you upon this eventful period of your lives. You now step into the ranks of the profession and claim its honors and its privileges. The honors acquired this evening are the result of study and labor; those of the future will depend on indefatigable study and the manner in which you use the knowledge acquired.

The fact is you have now attained that position in life where decision for the future will in all probability lead on to success and fortune or utter professional failure. Shakespeare has truely said, "There is a tide in the affairs of men, which taken at the flood leads on to fortune; omitted, all the voyage of their life is bound in shallows and in misery."

The more critically we examine the lives of men, the more deeply are we impressed with the fact that every act of our lives is a link in a chain which binds all together, and that from each act flows an influence which tells on successive acts. We may make each link in the chain of our lives good and strong for justice and the right, giving strength and stability to character, or we may make them weak and worthless so that the whole is rendered useless. Remember also, that one bad flawy link may destroy the usefulness of the best chain that can be made.

One small flaw in an otherwise good boiler may occasion great destruction to property and death to many. In like manner one bad act in the chain of our lives may impair the usefulness of the whole.

Physiology teaches us that there is a law in our organization which implies labor and rest. That even active elaboration and growth of an organ is followed by periods of quiet and rest. There are stages of physical energy and also mental, and however calm and tranquil may be the tides of our lives, still there are times when it is best to swim with the flood and press on to success.

We have stood on the ocean's shore, when wave after wave broke at our feet, and silently glided back into the waters, but presently there comes over this gentle ebb and flow a mountain billow riding over all these little waves; it surges shoreward and breaks itself high upon the beach. These are like the billows of our tide life, which even in the most calm and peaceful state stirs us from the depths of our being; and which, if taken at their full tide urges to action and may lead to fortune.

Founded on this great law of our being there are many maxims which seem to have a practical significance, for instance, "strike while the iron is hot," and "make hay while the sun shines," that is, take advantage of these openings and make the best use of them.

These diplomas are evidences that you have prepared yourselves for the professional duties you are about to assume. We take it for granted you fully understand your duties, to the profession and that neglect of these will or should ruin your professional prospects.

We regard it then as out of place to give you a formal lecture on dental science on this occasion; but we hope a few remarks on personal duties will not be deemed amiss.

The question is often asked why so few in our profession lay up a competency for advanced life. It is not because the most do not make enough during an ordinary life to secure this. While but few make large returns as in other professions, yet the most receive earlier returns or get into a living practice sooner.

Compared with medical practitioners and members of the legal profession, the first ten years of professional life will, as a general rule, give more lucrative remuneration, while after this the scales turn the other way.

In the practice of medicine and law, age and experience may claim and can receive something as compensation.

Now in making up our life work there are two or three elements which have not generally received that attention they deserve, and which placing ourselves on the level with the other professions should receive due consideration.

First the dentist must endure greater physical labor, he has to endure far more wear and tear of nervous power, even the general surgeon is not ordinarily taxed half as much. If he operates one or two hours per day he has sufficient time for rest and recuperation. But the dentist's labor lasts eight or more hours every day. This is why so few really old men are found in the practice of dentistry. We know there is a care and anxiety with the medical practitioner, so there must be with the dentist, but all this does not counterbalance the other, and besides there is much in the practice of medicine which secures change and out door exercises.

The legal profession have their forum and like all others, may over tax their vital powers; but the fact is the same measure of success in dental practice necessarily gives more to wear out the vital and physical energies than any other.

We wish you to start in your professional life with this one fact well understood which is that not one in ten can endure the labor and strain which a full practice will give for over twenty years.

The second consideration is, that no other profession is attended with the same necessary outlay.

Your instruments and materials are constant outlays, and the more you do the more you must spend in this way. Your fees must correspond to this outlay or you will become bankrupt before you know it. Your charity practice which is always incidental to the profession, is a source of outlay which is but little felt with others—this is always extra to that outlay for books and mental culture which is attached to all professions.

Good operations will not admit of much economy in the use of material. You perceive that the nature of your business forbids a general credit—you are always losing unless your cash receipts will meet your necessary outlay.

If you commence your professional life by loaning your services and material to the public, you will soon find that all your profits and more will be swallowed up in bad debts; and if one item of your service proves a failure, it will be an offset against a dozen successes—a dozen well filled teeth are mere quiet evidences of your skill, while one growling refractory organ disturbs the whole household. This growling member sometimes even stops payment for the twelve good ones.

It is said, that as a general rule, physicians' bills are more readily collected immediately after the recovery of the patient—you will find this equally applicable to your practice, and while there is much in the practice of dentistry which would seem to justify the prepayment of a part of a bill before the operation is completed at least with strangers, still it has been so repugnant to our sense of what is high-minded and honorable, that we could never establish such a rule. Some strangers with a sense of delicacy will make this offer, but they are such as you will always be most free to trust.

It will be hard sometimes to spend a day or two of hard work and twenty dollars in material, and never have your operation called for. It is worse than the clergyman who spent ten dollars in going to marry a couple, and only received three for his services.

But gentlemen, allow me to plan out a course of conduct which will be almost certain to insure success and a competency for the future.

My first lesson is, learn early to rely on your own skill and energy. Test at once your capacity for the best that is possible for your patient. Having selected your location in an intelligent community, a strict attention to your professional duties will soon secure the confidence of the public.

There is no better place to be found in office hours, than at your office.

It is not generally well to tack on to your profession some other business to fill up time or make a little more money.

If your profession will not pay for the use of all your energies, it will not for a part thereof. A man who attempts to make a living outside of his professsion, shows his own want of confidence in it, or at least a better relish for something else.

The second lesson, and sometimes a very difficult one to learn, is "live within your income." A great many of our apparent necessities are only so from our habits of indulgence. They should be regarded as luxuries well enough when they can be easily afforded, but not indispensable.

Many of our luxuries are not only unnecessary, but they are absolutely pernicious.

The drinking of ardent spirits and the use of tobacco possess not the first quality by which you will be the better prepared for the duties of life. Many young gentlemen spend fifty cents per day for these two luxuries, and scarce can pay their board bill. This is not the road to success.

Recollect gentlemen, habits are strong, and when formed in early life, they are apt to become our masters; be free, be honest. He who commences life determined to keep out of debt and lay up a little every year, will be his own master. The very economy to which he is forced at the start, becomes his greatest protection.

We can all afford to live within our means, but none beyond them. Let us look at what might be called a moderate and safe calculation for you all. The first two years you merely make a living and become established; then the third year you may lay up, say $200; the fourth year $300; the fifth year $500; the sixth year $600; the seventh year $700; the eighth year $800; the ninth year $900; the tenth year $1,000; total, $5,000. You are now well established in your profession, and for ten years to come should lay up a thousand dollars per year. This with the interest which has accumulated all this time, will put you in a position of independent competency.

Now if a kind providence has spared your life and health, you may and should nurse your somewhat exhausted nervous system and take life easy. We do not say retire from professional life, because some business has become essential to your health, comfort and happiness, and by this time you are too old to learn another. We have here sketched out what we believe to be perfectly practical in nine cases out of ten, when ordinary health is enjoyed. We know this is not the usual fortune of our profession, but it is not the fault of the profession; but it is attributable to expensive, reckless living and speculation. Usually when a few hundred dollars are laid by, the desire comes for some wonderful productive investment, something which will double up this little hoard every year without any labor at all, and oh, how many such openings are forced upon our attention.

When this dream of wealth is passed, the professional man wakes up to the sad discovery that he is not a good financier, and up to all the arts and devices of the trade.

In nine cases out of ten four per cent. bonds will prove a better investment. Gentlemen, we do not wish to weary you with this homely counsel, yet we feel that a somewhat long professional life has given us some experience in all these matters.

Solomon has a just reputation for wisdom, and he has given some most excellent advice to which we refer you. When you return to your homes and before you engage in the active duties of practice, open the Bibles you take with you as mementos of your alma mater, and turn to Proverbs, chapter xxii, 26th, 27th and 29th verses. Also chapter xi, 15th verse.

Before we conclude a few remarks on another subject will not be out of place. Most young gentlemen just through college, appear anxious to spend the first year or two in partnership with some one. These partnerships so often prove failures that we doubt their general propriety. But in conclusion, we will say that when your practice begins to pay, take at once a partner for life, and let the firm be husband and wife.

